# The impact of age on health utility values for older women with early-stage breast cancer: a systematic review and meta-regression

**DOI:** 10.1186/s12955-022-02067-w

**Published:** 2022-12-23

**Authors:** Yubo Wang, Sean P. Gavan, Douglas Steinke, Kwok-Leung Cheung, Li-Chia Chen

**Affiliations:** 1 Centre for Pharmacoepidemiology and Drug Safety, Division of Pharmacy and Optometry, School of Health Sciences, Faculty of Biology, Medicine and Health, The University of Manchester, Stopford Building, Oxford Road, 1stFloor Stopford Building, Manchester, M13 9PT UK; 2 Manchester Centre for Health Economics, Faculty of Biology, Medicine and Health, The University of Manchester, Oxford Road, Manchester, M13 9PL UK; 3 School of Medicine, University of Nottingham, Royal Derby Hospital Centre, Uttoxeter Road, Derby, DE22 3DT UK

**Keywords:** Early-stage breast cancer, Economic evaluation, Health state utility values, Meta-regression, Older women, Systematic review

## Abstract

**Introduction:**

An increasing number of postmenopausal women are diagnosed with breast cancer at an older age (≥ 70 years). There is a lack of synthesised health utility data to support decision-making for managing breast cancer in this older population. This study aimed to identify the availability of, and the subsequent impact of age on, health state utility values (HSUVs) measured by the EQ-5D for older women with early-stage breast cancer.

**Method:**

This systematic review identified EQ-5D (3L or 5L version) HSUVs for postmenopausal women with early-stage breast cancer. Studies were identified from a previous systematic review (inception to 2009) and an electronic database search (Medline and Embase; 2009 to September 2021). Mean HSUVs were summarised by health state. Quality appraisal was performed on studies reporting HSUVs for older ages (≥ 70 years). Multivariable meta-regression assessed the association between HSUVs and age, health state, treatments received, and time of measuring the utility values (greater or less than one year post-treatment).

**Results:**

Fifty EQ-5D HSUVs were identified from 13 studies. Mean HSUVs decreased as health state worsened: from the stable (mean=0.83) to progression (mean=0.79) and advanced (mean=0.68) states. Two studies reported six HSUVs estimated from the sample of women with a mean age ≥ 70. Meta-regression model fit improved by including age as an independent variable and attenuated the estimated utility decrements associated with worse health states. Utility decrements for the progression and advanced states were -0.052 (95%CI: -0.097, -0.007) and -0.143 (95%CI: -0.264, -0.022) respectively. The breast cancer-specific utility decrement associated with a one-year increase in age was -0.001 (95%CI: -0.004, 0.002).

**Conclusion:**

Relevant and accurate HSUVs are essential to help support decision-making about the most effective and cost-effective ways to manage early-stage breast cancer in older women. Age has a vital role in determining health utility values in this population. This study provides analysts and decision-makers with HSUVs and utility decrements that reflect the disease process in this older population.

**Supplementary Information:**

The online version contains supplementary material available at 10.1186/s12955-022-02067-w.

## Introduction

Health state utility values (HSUVs) quantify preference for specific health states and are a vital source of evidence for health economic evaluations to inform resource allocation decisions and treatment recommendations [1]. Best practice guidance explains how the most relevant HSUVs to inform decision-making should reflect the health characteristics of the target patient population [2, 3]. To improve the accuracy of HSUVs for specific populations, there is a growing focus on investigating how the impact of age is quantified across different health conditions [4]. The incidence of health conditions, such as breast cancer, is starting to increase in older patients due to an ageing population [5]. In light of this trend, there is a need to improve the robustness of HSUV estimates and strengthen the evidence base that will support treatment recommendations in these older patient populations.

The quality of life of women with breast cancer varies with different factors. The HSUVs used in economic modelling must reflect the target population's relevant disease health states, treatments received, and patient characteristics [6]. Age is a crucial risk factor influencing the incidence and treatment of female breast cancer [7]. One-third of new breast cancer cases in England were diagnosed at an older age (> 70 years) [8]. Older age typically corresponds with lower HSUVs due to weaker physical functioning and multimorbidity [9, 10]. However, there are few health economic evaluations for older women with breast cancer that used HSUVs measured directly from patients aged 70 years or more.

In 2022, a systematic review identified seven economic evaluations of breast cancer treatments for older women [11]. Most studies in this review (*n* = 6; 86%) sourced health utility data from patients younger than 70 years, and adjusted these estimates to correspond with an older population. A better understanding of the health utility values available in this growing patient population will be valuable to support the need for economic evidence designed to inform the management of older women with early-stage breast cancer.

A systematic review and meta-regression by Peasgood et al*.* (2010) [12] synthesised health utility values for early-stage and metastatic breast cancer. Similarly, Kaur et al*.* (2022) [13] report a meta-regression of health utility values across different stages of breast cancer and treatment. Both studies demonstrate the value of meta-regression to establish whether patient-level and treatment-related variables are associated with mean HSUVs. Although these analyses included several variables associated with health utility (for example, disease health state, treatment, and HSUV valuation method), age was not included as an independent variable in either meta-regression. This specification may overestimate the health utility decrement associated with disease progression. To improve the usefulness of these estimates for generating future economic evidence, including age as an independent variable within a meta-regression will help to estimate its impact on HSUVs for older women with breast cancer.

This study aimed to identify the availability of, and the subsequent impact of age on, HSUVs measured by EQ-5D for older women with early-stage breast cancer. To achieve this aim, there were three objectives: (1) identify studies that estimated HSUVs by EQ-5D in a sample of postmenopausal women with early-stage breast cancer; (2) describe and appraise the quality of HSUV estimates in the subgroup of studies that focussed on older women (aged ≥ 70 years); and (3) evaluate how age affects the statistical association between HSUVs and other relevant variables.

## Method

A systematic review to identify all published studies reporting HSUVs for postmenopausal women with early-stage breast cancer was conducted according to the Preferred Reporting Items for Systematic Reviews and Meta-analyses (PRISMA 2020) [14] (Supplementary Appendix 1). The protocol for this systematic review is registered at PROSPERO (no. CRD42021232743). After registration, a minor revision was made to only include studies that measured HSUVs by an EQ-5D instrument only, to avoid duplication with another systematic review by Kaur et al. published in 2022 [13]. EQ-5D is the generic multi-attribute measure of health status used most often by health technology assessment bodies around the world. Hence, focusing on the EQ-5D instrument ensures that this study is valuable for health care decision-makers [15].

### Inclusion and exclusion criteria

Studies were included if they (i) reported an original HSUV for a specific health state for postmenopausal women with breast cancer e.g., stable (defined as cancer that does not worsen after treatment, or diagnosed as stage I or II), progressed (tumour locally spread or diagnosed as stage III), or advanced disease states (tumour distant metastases or diagnosed as stage IV), (ii) measured using an EQ-5D instrument (EQ-5D-3L or EQ-5D-5L) and valued with a tariff that is used routinely for decision-making, and (iii) were written in English (Table 1). Postmenopausal women as the target population were initially identified by whether the study self-reported the term “postmenopausal women” or not. If not, the cut-off age ≥ 45 years was used to define post-menopause, according to the National Health Service (NHS) in England [16].

**Table 1:** Inclusion and exclusion criteria for the study

Component	Inclusion criteria	Exclusion criteria
Population and conditions	Postmenopausal women with (operable, Stage I, Stage II, or early stage) breast cancer	• Only premenopausal women • Only male breast cancer • Only metastatic breast cancer • Unconfirmed breast cancer • Other diseases
Intervention & Comparator	Any intervention for breast cancer	No restriction on the intervention
Outcome	Study reported at least one original utility value measured by EQ-5D (3L or 5L)	• No original utility value reported • Unspecified/not clearly specified health states relating to breast cancer • Psychometric validation studies • Description of health states without interval properties rather than the valuation of health states • EQ-5D-5L England tariff
Language	English	Other languages without English translation
Publication	Full-text article	Conference abstract or proceeding, abstract without full article, letter to editors, editorial, commentary, and news

The criteria for inclusion and exclusion were based on the PICO framework [17]

### Literature search

Relevant studies that met the inclusion criteria were identified in two stages. In the first stage, studies published from inception to 2009 were identified from the systematic review by Peasgood et al*.* (2010) [12]. The review by Peasgood et al*.* (2010) [12] comprehensively searched thirteen databases to identify HSUVs for breast cancer measured using preference-based instruments, and also using Google Scholar as a supplementary data source to identify the target literature. The search strategies in the review by Peasgood et al*.* (2010) [12] were developed from a previously published systematic review by Hind et al*.* (2010) [18] for early breast cancer. These two reviews have informed the evidence base for earlier National Institute for Health and Care Excellence (NICE) clinical guidelines and are highly cited in other published reviews or original studies [19–24]. Therefore, the review by Peasgood et al*.* (2010) was considered to be a good data source for identifying the studies reporting HSUVs in breast cancer before 2009. From this initial set of references, studies that reported HSUVs measured using an EQ-5D instrument were identified and retrieved for full text review.

In the second stage, studies published from 2009 until 21 September 2021 were identified from electronic medical databases by applying structured search strategies to Ovid MEDLINE® and Epub Ahead of Print, In-Process & Other Non-Indexed Citations, Daily and Versions ® 2009 January to 2021 22 September and Ovid EMBASE® from 2009 January to 2021 22 September. The search strategies (Supplementary Appendix 2) included relevant terms for breast cancer used by Peasgood et al*.* (2010) [12] and HSUVs. Terms to identify HSUVs were sourced from the electronic database search filters reported by the Centre for Reviews and Dissemination [25].

### Study selection

The titles and abstracts of studies identified from the electronic database search were screened independently by three investigators (SB, MA, YW) against the inclusion criteria. The concordance between reviewers was calculated by three pairwise intra-class correlation coefficients (ICC) [26]. ICC values less than 0.50, between 0.50 and 0.75, 0.75 and 0.90, and greater than 0.90 indicate poor, moderate, good and excellent reliability, respectively [27]. Three investigators (SB, MA, YW) independently reviewed the full text of eligible studies. Discrepancies were resolved through consensus with other reviewers (SG & LCC) to finalise the selection of studies. This was done to ensure that the reviewers appropriately applied the inclusion and exclusion criteria in the screening process.

### Data extraction

Data were extracted from the included studies independently by three reviewers (SB, MA, YW) using a pre-designed data collection form and then merged by YW for analysis. Extracted data included three sections: (1) characteristics of the study, i.e., the author, year and country of the study; (2) methods of health utility valuation, i.e., mean age of estimation sample, instrument to measure health utility values (EQ-5D-3L or EQ-5D-5L), the valuation tariff, and the sample size of the study; and (3) estimated health utility values for specific health states (stable, progression and advanced state), i.e., mean utility value, standard deviation (SD), standard error (SE), interquartile range (IQR), or 95% confidence interval (95%CI). Studies that estimated utility values with the EQ-5D-5L tariff for England [28] were excluded because the National Institute for Health and Care Excellence (NICE) does not recommend using the tariff due to concern about data collection and analysis methods [29]. In such circumstances, studies that estimated UK EQ-5D-3L utility values from EQ-5D-5L profiles by a recommended mapping method were included [30].

### Data synthesis

Descriptive statistics were first used to present the included studies, study characteristics and the mean (SD or SE), median (IQR) and the range (or 95%CI) of the HSUVs. These results were summarised narratively, presented graphically, and stratified by different health states and treatments where possible for the full sample of postmenopausal women. For studies that did not report the SD, the estimated SD was calculated from the mean value, sample size, SE or 95%CI if necessary, based on the method suggested by the Cochrane Library [31].

The subgroup of studies which estimated HSUVs using a sample of older women (mean age ≥ 70 years) were described by the study design, country, mean age of respondent, elicitation method and quality appraisal. As there are no agreed criteria to appraise the quality of HSUVs [26], four questions (in Table 3) were used to appraise the quality of the studies that estimated HSUVs from an older population. These four questions were identified from an appraisal tool (including 17 questions) developed by Nerich et al. (2017) [32] (Full appraisal tool in Appendix 3). According to a systematic review of HSUV appraisal tools by Zoratti et al. [33], these four questions from the tool developed by Nerich et al. (2017) [32] were useful to appraise the quality of breast cancer HSUVs. YW independently appraised the quality of studies, and the results of the appraisal were categorised as yes (complete), yes (partial), no, and not assessable. Publication bias for HSUVs is difficult to determine because they are usually reported as secondary outcomes. Thus, publication bias in this review was not assessed. 

 The HSUVs were synthesised by a meta-regression following the methods used by Peasgood et al*.* (2010) [12] to identify the association between HSUVs and different independent variables. A linear regression model was used with the mean HSUV from each study as the dependent variable. Age is a critical factor that influences HSUVs. Therefore, this study compared the results from two regression specifications. The first specification included the reported mean age of the estimation sample for each HSUV as a continuous independent variable. The second specification omitted the reported mean age from the set of independent variables. The performance of these two specifications was compared using the coefficient of determination (R^2^) to assess the goodness of fit [34].

According to Peasgood et al.’s review [12], several additional variables that may influence the HSUV measurement and valuation were included in the analysis: disease health state, the instrument to measure health utility, treatment received, and valuation time. Disease health state (stable, progressed disease, or advanced disease states), instrument to measure health utility (EQ-5D-3L or EQ-5D-5L), treatment received (surgery, surgery alone with adjuvant therapies, or unspecified treatment), and valuation time (less or more than one year after diagnosis) were measured as categorical variables. ‘Surgery’ comprised different types of surgical intervention (for example, mastectomy or breast conserving surgery) to reduce the number of independent variables in the meta-regression, following the approach by Kaur et al*.* [13].

Other study characteristics (e.g., country of the study, valuation tariff, trial or observational study design, intervention and comparators in the study) were not included as independent variables in the meta-regression. Given the sample size of the meta-regression, this decision was made to prevent collinearity between categorical independent variables. The regression model weighted by the inverse of the SD for each HSUV. This approach gives greater weight to HSUVs values with a smaller SD because they offer better precision in the true utility value than those with a larger SD. Cluster-robust standard errors were used to account for within-study correlation because some studies contributed more than one HSUV to the meta-regression which were likely to be correlated with each other [35]. The meta-regression was performed using Stata 14.0 (Stata Corp, College Station, TX) [36].

## Results

### Selection of studies

**Fig. 1:**
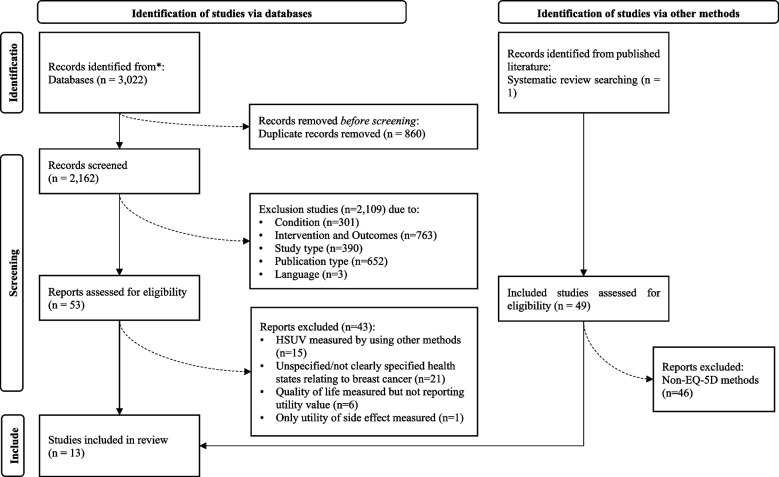
PRISMA flow diagram for selection of studies

Forty-nine potentially eligible articles were identified from the systematic review by Peasgood et al*.* [12], and 3,022 articles were identified from the electronic medical database search (Fig. 1). Thirteen studies met the inclusion criteria and were included in the systematic review. The reasons for exclusion are summarised in Fig. 1 and the supplement file (Supplementary Appendix 4). The ICC value indicated good and excellent reliability between reviewers (pairwise ICCs between three reviewers were: 0.78, 0.89 and 0.96).

### Study characteristics

**Fig. 2:**
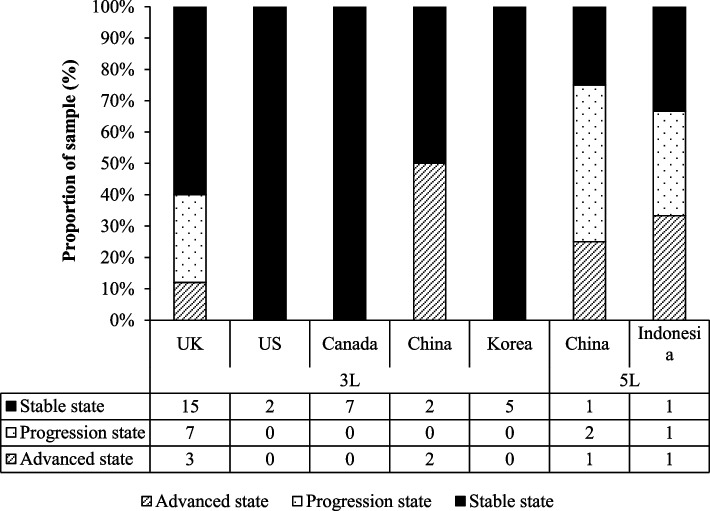
Health state utility values by health state and valuation tariff

Fifty HSUVs were identified from the 13 studies [37–49] (Table 2). The HSUVs were distributed across three health states: stable (*n* = 33), progressed disease (*n* = 10), and advanced disease (*n* = 7). The EQ-5D-3L (*n* = 43) [37–46, 48] instrument was used more often than the EQ-5D-5L instrument (*n* = 7) [47, 49]. Six different valuation tariffs were applied across the sample, including the UK 3L (*n* = 28) [37–39, 41, 42, 46, 48], US 3L (*n* = 2) [40], Canada 3L (*n* = 4) [45], Korea 3L (*n* = 5) [44], China 3L (*n* = 4) [43], China 5L (*n* = 4) [47], and Indonesian 5L (*n* = 3) [49] tariffs (Fig. 2). Across the whole sample, these HSUV values were estimated from patients with a mean age between 44 and 75 years. One study defined their sample as 'postmenopausal women [49], and the remaining studies (92%) had a sample of women whose mean age was over 45 years.

**Table 2:** Characteristics of identified studies (*n* = 13)

Author	Country	Study period	Study type	Respondent	Method of valuation	Valuation Tariff	Mean Age	Sample size
***EQ-5D-3L***								
Conner-Spady, et al. (2005) [37]	Canada	04/1995–10/1998	Questionnaire	Patients' own health	EQ-5D-3L	UK	44.7	52
Lidgren, et al. (2007) [38]	Sweden	04–05/2005	Questionnaire	Patients' own health	EQ-5D-3L	UK	57	345
Kimman, et al. (2009) [39]	Netherland	07/2005–09/2007	Questionnaire	Patients' own health	EQ-5D-3L	UK	55.8	192
Freedman*, *et al*.* (2010) [40]*	USA	2010	Questionnaire	Patients' own health	EQ-5D-3L	US	45–64	1050
Williams, et al. (2011) [41]	UK	1997	Questionnaire	Patients' own health	EQ-5D-3L	UK	72.8	255
Yousefi*, *et al*.* (2016) [42]	Iran	11/2013–06/2014	Questionnaire	Patients' own health	EQ-5D-3L	UK	46.7	163
Wang*, *et al*.* (2018) [43]	China	12/2016–03/2017	Questionnaire	Patients' own health	EQ-5D-3L	China	49.1	2828
Yu*, *et al*.* (2018) [44]	Korea	01/2012–06/2012	Questionnaire	Patients' own health	EQ-5D-3L	Korea	48.9	226
Sattar*, *et al*.* (2019) [45]	Canada	10/2014–10/2015	Questionnaire	Patients' own health	EQ-5D-3L	Canada	75.3	58
Tanaka*, *et al*.* (2019) [46]**	Japan	Not stated	Questionnaire	Patients' own health	EQ-5D-3L	UK	53.4/57.6	38
Zigman*, *et al*.* (2020) [48]	Croatia	01/2016–12/2016	Questionnaire	Patients' own health	EQ-5D-3L	UK	44.7	114
***EQ-5D-5L***								
Yang*, *et al*.* (2020) [47]	China	08/2017–05/20	Questionnaire	Patients' own health	EQ-5D-5L	China	51.37	446
Etikasari*, *et al*.* (2021) [49]	Indonesia	01/2019–08/2019	Questionnaire	Patients' own health	EQ-5D-5L	Indonesian	59.2	126

^*^ Respondent age in Freedman [40] was 45–64 years (57%);

^**^ Respondent age in Tanaka [46] for usual care: 53.4 years, pharmacist care: 57.6 years

**Fig. 3:**
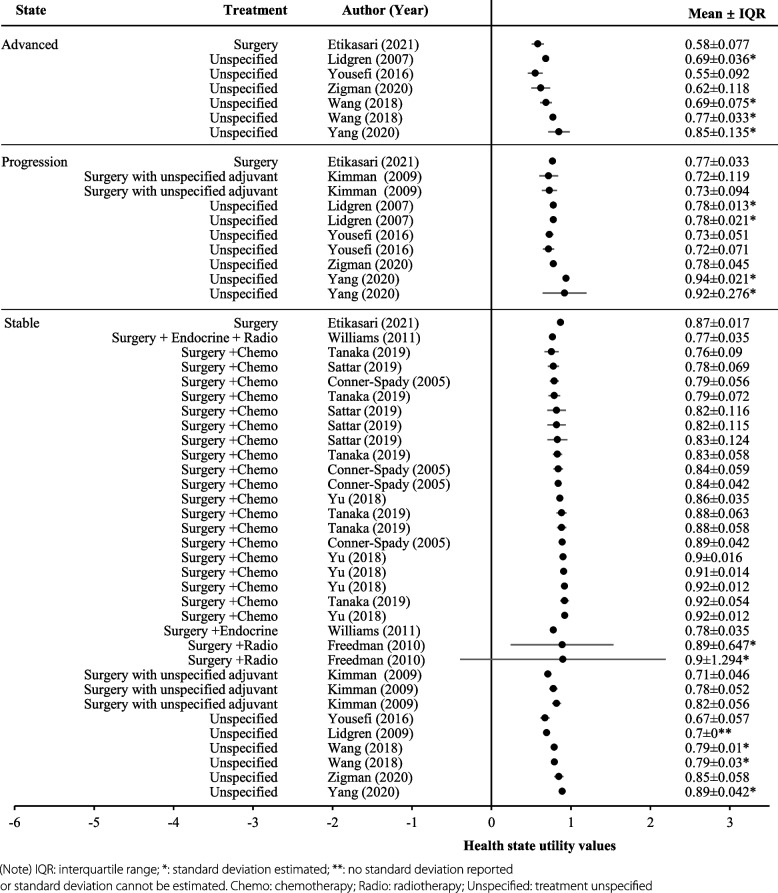
Health state utility values by health state

**Fig. 4:**
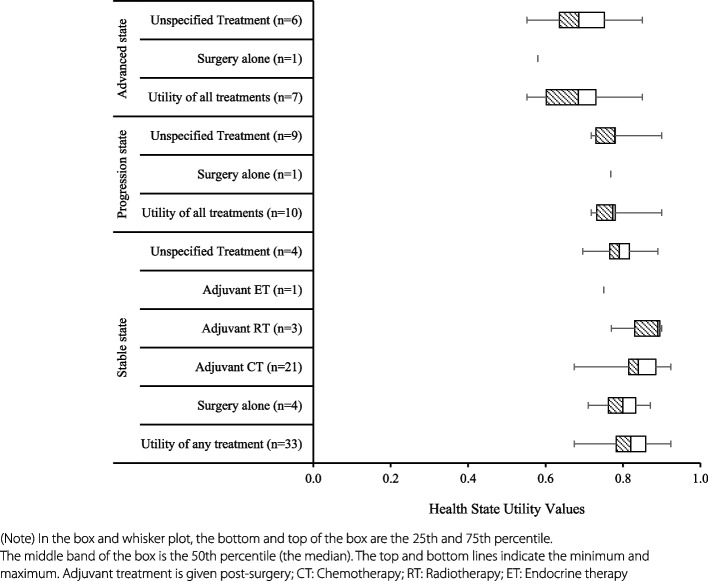
Utility values for three health states stratified by treatment

The subset of health utility values for the stable state (*n* = 33, same mean and median: 0.83; range: 0.67 to 0.92) were higher than the progressed disease state (*n* = 10, mean: 0.79; median: 0.77; range: 0.72–0.94) and advanced disease state (*n* = 7, mean: 0.68; median: 0.69; range: 0.55–0.85) (Fig. 3). Figure 4 (a box-and-whisker plot) reports the distribution of HSUVs by disease state and treatment received. Of the 33 utility values for the stable health state, treatment was not specified for six utility values (mean: 0.78; median: 0.79; range: 0.67-0.89). Patients who received surgery with adjuvant radiotherapy had the highest utility value (*n* = 3; mean: 0.86; median: 0.89; range: 0.78–0.90), followed by surgery with adjuvant chemotherapy (*n* = 19; mean: 0.85, median: 0.84; range: 0.76-0.92) and surgery alone (*n* = 1) or with unspecific adjuvant treatment (*n* = 3; same mean and median: 0.80; range: 0.71–0.87).

It was impossible to stratify HSUVs by treatment for progressed and advanced health states, as only one HSUV specified treatment with surgery alone in both the progressed state (0.77) and advanced disease state (0.58). The remaining values for these two health states were not attached to a specific treatment. The mean of these remaining HSUVs was 0.79 for the progressed state (*n* = 9; median: 0.78; range: 0.72–0.94), and 0.69 for the advanced state (*n* = 6; median: 0.69; range: 0.55–0.85) (Fig. 4). (Detailed mean utility values extracted in each study reported in Appendix 5).

### Quality appraisal of studies measuring HSUV in older women

There were 6 HSUVs for the stable disease state estimated specifically from a sample of older patients (mean age ≥ 70 years) in two clinical trials by Williams et al. (2011) [41] (*n* = 2) and Sattar et al. (2019) [45] (*n* = 4). These two studies are now described in further detail. The quality appraisal criteria are reported in Table 3.

**Table 3:** Quality appraisal of two studies for older women

**No**	**Questions**	**Williams et al*.* (2011) ** [41]	**Sattar et al*.* (2019) ** [45]
E1	Is an explanation provided for the choice of technique(s) used to elicit HSUVs?	Complete	Partial
E2	Is a comprehensive description provided of technique(s) used to elicit the obtained HSUVs?	Complete	Complete
E3	Is an explanation provided for the choice of the population used to elicit HSUVs (i.e., patient, healthcare professional [and type], expert, general population)?	Partial	Partial
E4	Is a comprehensive description provided for the population used to elicit HSUVs (i.e., characteristics, size, and nationality)?	Complete	Complete

Complete: Yes (complete); Partial: Yes (partial); E: elicitation

Appraisal questions extracted from the study by Nerich et al*.* (2017) [32]

Williams et al*.* (2011) [41] conducted a clinical trial in the UK with 248 older participants (mean age: 72 years, SD: 5) who had primary breast cancer and received surgery or adjuvant endocrine therapy with or without radiotherapy. The duration of follow-up was five years. The EQ-5D-3L instrument and UK tariff [50] was used to estimate the HSUVs. Across both arms, 12 HSUVs in total were estimated at baseline, and 3.5, 9, 15, 36, and 60 months after surgery. The specific health state associated with these HSUVs was not reported. Assuming that patients were stable within 6 months after surgery, at 3.5 months, the HSUVs for adjuvant endocrine therapy alone was 0.77 (95%CI: 0.74 to 0.80), and for adjuvant endocrine therapy plus radiotherapy was 0.78 (95%CI: 0.75 to 0.81).

Sattar et al. (2019) [45] conducted a clinical trial in older participants with breast cancer who received surgery and adjuvant chemotherapy with (*n* = 30, mean age: 75 years) and without (the usual care; *n* = 28, mean age: 75 years) a geriatric assessment in Canada. The EQ-5D-3L instrument and Canadian tariff [51] was used to estimate the HSUVs. Across both arms, eight HSUVs were estimated at baseline and 3, 6, and 12 months. The specific health state associated with these HSUVs was not reported. Assuming that patients were stable within 6 months after surgery, the median HSUVs for patients with the geriatric assessment at 3 and 6 months were 0.82 (IQR: 0.29), and 0.82 (IQR: 0.27), respectively. The median HSUVs for patients without the geriatric assessment at the same time periods were 0.78 (IQR: 0.15), and 0.83 (IQR: 0.22).

Both two studies completed or partially reported four questions of the quality appraisal tool (Table 3). Williams et al*.* (2011) [41] reported the reason for selecting the EQ-5D-3L instrument to measure the HSUVs was due to the recommendations by the NICE reference case, and fully explained the reason to use the EQ-5D-3L UK valuation tariff. Both studies [41, 45] fully reported details about characteristics of study population as they are randomised control trials. Therefore, the two studies [41, 45] are high quality studies based on this quality appraisal tool.

### Regression analysis

Table 4 reports the results of the meta-regression analyses. The specification that included age as an independent variable had a better goodness of fit (R^2^ increased from 0.686 to 0.691). Across all model specifications, the variables for disease health state, treatment, and instrument to measure HSUVs had a statistically significant (*p* < 0.05) association with the mean HSUV. Age was estimated to have a negative but non-statistically significant coefficient (-0.001, 95%CI: -0.004 to 0.002). This result indicates that expected HSUVs reduce as postmenopausal women with breast cancer become older. The statistically significant and negative coefficients on progression (-0.052) and advanced disease states (-0.143) indicated that expected HSUVs reduce as disease worsens. Compared with surgery alone, adjuvant treatments improved the mean HSUVs with an increment of 0.205 for adjuvant chemotherapy, 0.200 for adjuvant radiotherapy, and 0.085 for adjuvant endocrine therapy. The HSUV for patients over one year after treatment was 0.045 units higher than those who received treatment within one year.

**Table 4:** Regression models for HSUVs

Variables	Estimated coefficient ± 95% CI			
	**Age-adjusted**		**No age adjustment**	
	**Coefficient (95% CI)**	***p* value**	**Coefficient (95% CI)**	***p* value**
**Age**	-0.001 (-0.004, 0.002)	0.502	-	-
**Health state reference: stable state (*n* = 32)**				
Progressed state (*n* = 10)	-0.052 (-0.097, -0.007)	0.027	-0.055 (-0.102, -0.008)	0.021
Advanced state (*n* = 8)	-0.143 (-0.264, -0.022)	0.02	-0.146 (-0.267, -0.024)	0.055
**Instrument reference: EQ-5D-3L instrument (*n* = 43)**				
EQ-5D-5L (*n* = 7)	0.176 (0.115, 0.237)	< 0.001	0.176 (0.120, 0.233)	0.025
**Treatment reference: surgery alone (*n* = 3)**				
Surgery adjuvant chemotherapy (*n* = 20)	0.205 (0.133, 0.277)	< 0.001	0.209 (0.144, 0.274)	0.029
Surgery adjuvant radiotherapy (*n* = 2)	0.200 (0.141, 0.259)	< 0.001	0.204 (0.157, 0.252)	0.021
Surgery adjuvant endocrine therapy (*n* = 5)	0.085 (0.036, 0.135)	0.003	0.085 (0.040, 0.131)	0.020
Surgery without specified adjuvant (*n* = 19)	0.107 (0.069, 0.144)	< 0.001	0.114 (0.084, 0.144)	0.013
Unspecified treatment (*n* = 1)	0.148 (0.081, 0.215)	<0.001	0.136 (0.072, 0.200)	0.029
**Valuation time reference: less than one year (*n* = 19)**				
Over 1 year (*n* = 31)	0.045 (0.006, 0.083)	0.027	0.050 (0.012, 0.088)	0.017
Constant	0.696 (0.485, 0.908)	< 0.001	0.639 (0.575, 0.703)	0.029
Observations	50		50	
**R-squared**	0.691		0.686	

## Discussion

This study provides a valuable set of utility values for older women with early-stage breast cancer to support future economic analyses and decision-making. Six utility values for patients with stable breast cancer, measured HSUVs from an older population with mean age ≥ 70 years, were identified from two studies conducted in the UK [41] and Canada [45]. In addition, the meta-regression quantified the disease-specific age-related utility decrement for older women with breast cancer and provided improved estimates of HSUV modifiers for age by controlling for disease state and treatment. Collectively, these estimates improve the robustness of evidence for future quality of life research and health economic evaluations for older women with breast cancer.

There is consensus among healthcare providers that the quality of life for women with breast cancer reduces with ageing due to comorbidity and frailty related to poor physical functioning [52]. Therefore, it is necessary to incorporate this reduction of health utility within economic evaluations to improve the robustness of quality-adjusted life year (QALY) estimates [2]. The association of HUSVs with other key factors, including treatment types (e.g., mastectomy or non-specified surgery type, adjuvant chemotherapy or radiotherapy), valuation methods (e.g., EQ-5D, standard gamble, time trade-off), and valuation respondents (patients, clinicians or scenario), has been assessed by previously published studies (Peasgood et al*.* (2010) [12] and Kaur et al*.* (2022) [13]). Our study quantified the association between HSUVs and age by controlling for similar variables. The results of the meta-regression in our review provide insights for health care analysts undertaking future research to improve decision-making for breast cancer management in older women.

For healthcare decision-makers who use health economic evidence, decisions are made according to the incremental expected cost and health benefits of care irrespective of whether differences are statistically significant [53]. Therefore, although the association between age and HSUVs had no statistical difference in our analysis, the finding is still informative for health care decision-making. First, the catalogue of EQ-5D values by Sullivan et al. [50] estimated an age-related utility decrement of -0.0003 in the general population. However, the results from this study indicate that the condition-specific age-related utility decrement for breast cancer has a larger magnitude (-0.0013) than for the general population. The validity of future studies designed to estimate the lifetime trajectory of HSUVs may improve by using condition-specific age-related utility decrements (as part of the base case or sensitivity analysis) instead of those values estimated from the general population. Second, the utility decrement associated with disease progression may be overestimated by omitting age as an independent variable (for example, compare the utility decrements for disease states across both regression specifications in Table 4). In comparison with other published results, the utility decrement of the progressed state compared with the stable state was -0.143 in Peasgood et al. [12] and -0.0549 in the present study. Similarly, the utility decrement of the advanced state was -0.338 in Peasgood et al. [12] and -0.1521 in the present study. There are two main reasons to explain the differences between these estimated decrements. First, the review by Peasgood [12] included values measured using various preference-based instruments, while we only included HSUVs measured by EQ-5D. Second, Peasgood [12] analysed women with breast cancer in all age groups, whereas this review focused on postmenopausal women with early-stage breast cancer. Collectively, these reasons led to a smaller sample size for the meta-regression compared with other published examples. Consequently, the results from the regression model in the present study provide relevant HSUV decrements for postmenopausal women with early-stage breast cancer for decision-makers who use an EQ-5D instrument.

In addition, a growing phenomenon in managing breast cancer for older women is that many patients will receive primary endocrine therapy, instead of surgery, as their initial treatment [54–56]. Yet this review found no studies that estimated HSUVs for women with early-stage breast cancer who received non-surgical first-line treatment. Instead, the identified studies comprised patients who received surgery with or without adjuvant treatment. One study did not meet the inclusion criteria for this review (because HSUVs were measured using the EQ-5D-5L England tariff) but did measure HSUVs for older women receiving primary endocrine therapy [57]. The size of the patient cohort who receive non-surgical intervention in clinical practice is likely to increase, all else being equal, as the population ages and more breast cancer cases are diagnosed at a later age [55, 56]. A greater focus on estimating health utility values for this patient cohort will be valuable to better understand how HSUVs can be affected by the direct impact of treatment-related side effects and the longer-term impact of changes in disease outcomes.

One limitation of this review was related to the search process. The search strategy only identified published manuscripts from peer-reviewed academic journals and may have missed HSUVs reported in the grey literature and other data sources. However, the results indicate that the sample of included studies may be potentially sufficient to pool and quantify the condition-specific association between age and health utility for older women with breast cancer. Searching Medline and Embase has a high ability to identify relevant studies (Bramer et al. [58] report a 92.8% recall rate) and have been used effectively by other systematic reviews of HSUVs [59].

A second limitation was that only HSUVs measured by the EQ-5D instruments were included in the analysis. This may constrain the generalisability of the results because the estimated associations are not likely to apply to other preference-based instruments (such as the Short Form-6 Dimension [60] or the Health Utilities Index [61]). However, the focus on EQ-5D instruments will be most valuable to health care decision-makers because of its widespread global use by health technology assessment bodies [15]. Finally, omitting the EQ-5D valuation tariff as an independent variable in the meta-regression is a limitation if these cross-country differences impacted the estimated mean HSUV. This impact could be explored further as more HSUVs for older women with breast cancer become available across different countries in the future.

Future research can aim to investigate the impact of age on HSUVs estimated by other preference-based instruments for older women with breast cancer, and identify studies from other data sources to supplement the current results. In addition, future studies can be designed to establish whether HSUVs estimated by EQ-5D instruments are affected by the treatment received once older patients enter the progressed or advanced disease states. Finally, other chronic conditions (such as diabetes and cardiovascular disease) are becoming more common due to an ageing population [62]. Future studies can estimate the condition-specific age-related utility decrement for different diseases to improve the validity of lifetime HSUV estimates and the quality of evidence that informs health care decision-making.

## Conclusion

This study strengthens the HSUV evidence base to help inform future decision-making regarding older women with breast cancer. Analysts can use the data sources presented in this review to identify age-specific HSUV estimates that are most relevant for their decision-making context. The age-adjusted health utility decrements for disease states can improve the quality of crucial input parameter values for cost-effectiveness analyses of treatments for this older population. The estimated condition-specific health utility decrement will improve the validity of lifetime HSUV estimates for people with breast cancer. A greater emphasis on accounting for the impact of age on HSUVs will improve the robustness of evidence essential to guide health care decision-making for the growing number of older patients diagnosed with early-stage breast cancer.

## Supplementary Information

**Additional file 1. **Appendix 1. PRISMA 2020 Checklist. Appendix 2. Search Strategy for Databases. Appendix 3. Quality Appraisal Tool. Appendix 4. Reasons for Excluding Studies during Database Record Screening. Appendix 5. Data Extraction of the Mean Utility Values. 

## Data Availability

Supplement information is included in the appendices.
