# Supplementary material for: The impact of age on health utility values for older women with early-stage breast cancer: a systematic review and meta-regression
Source: Health Qual Life Outcomes. 2022 Dec 23;20:169. doi: 10.1186/s12955-022-02067-w (PMC9789668; doi:10.1186/s12955-022-02067-w)
Supplement: Supplementary file 1 — Additional file 1. Appendix 1. PRISMA 2020 Checklist. Appendix 2. Search Strategy for Databases. Appendix 3. Quality Appraisal Tool. Appendix 4. Reasons for Excluding Studies during Database Record Screening. Appendix 5. Data Extraction of the Mean Utility Values. [file 12955_2022_2067_MOESM1_ESM.docx]

**The Impact of Age on Health Utility Values for Older Women with Early-stage Breast Cancer: A Systematic Review and Meta-regression**

**Supplementary Appendix**

Appendix 1. PRISMA 2020 Checklist (page 2)

Appendix 2. Search Strategy for Databases (page 6)

Appendix 3. Quality Appraisal Tool (page 8)

Appendix 4. Reasons for Excluding Studies during Database Record Screening (page 9)

Appendix 5. Data Extraction of the Mean Utility Values (page 10)

**Supplementary files:**

**Appendix 1. PRISMA 2020 Checklist**

| **Section and Topic** | **Item #** | **Checklist item** | **Location where item is reported** |
| --- | --- | --- | --- |
| **TITLE** | | |  |
| Title | 1 | Identify the report as a systematic review. | Title |
| **ABSTRACT** | | |  |
| Abstract | 2 | See the PRISMA 2020 for Abstracts checklist. | Abstract |
| **INTRODUCTION** | | |  |
| Rationale | 3 | Describe the rationale for the review in the context of existing knowledge. | Introduction |
| Objectives | 4 | Provide an explicit statement of the objective(s) or question(s) the review addresses. | Introduction: the final paragraph |
| **METHODS** | | |  |
| Eligibility criteria | 5 | Specify the inclusion and exclusion criteria for the review and how studies were grouped for the syntheses. | Method: Inclusion and exclusion |
| Information sources | 6 | Specify all databases, registers, websites, organisations, reference lists and other sources searched or consulted to identify studies. Specify the date when each source was last searched or consulted. | Method: Literature search |
| Search strategy | 7 | Present the full search strategies for all databases, registers and websites, including any filters and limits used. | Method: Literature search |
| Selection process | 8 | Specify the methods used to decide whether a study met the inclusion criteria of the review, including how many reviewers screened each record and each report retrieved, whether they worked independently, and if applicable, details of automation tools used in the process. | Method: Study selection |
| Data collection process | 9 | Specify the methods used to collect data from reports, including how many reviewers collected data from each report, whether they worked independently, any processes for obtaining or confirming data from study investigators, and if applicable, details of automation tools used in the process. | Method: Data extraction |
| Data items | 10a | List and define all outcomes for which data were sought. Specify whether all results that were compatible with each outcome domain in each study were sought (e.g. for all measures, time points, analyses), and if not, the methods used to decide which results to collect. | Method: Data extraction |
|  | 10b | List and define all other variables for which data were sought (e.g. participant and intervention characteristics, funding sources). Describe any assumptions made about any missing or unclear information. | Method: Data extraction |
| Study risk of bias assessment | 11 | Specify the methods used to assess risk of bias in the included studies, including details of the tool(s) used, how many reviewers assessed each study and whether they worked independently, and if applicable, details of automation tools used in the process. | Method: Data synthesis, the 2^nd^ paragraph |
| Effect measures | 12 | Specify for each outcome the effect measure(s) (e.g. risk ratio, mean difference) used in the synthesis or presentation of results. | Method: Data synthesis |
| Synthesis methods | 13a | Describe the processes used to decide which studies were eligible for each synthesis (e.g. tabulating the study intervention characteristics and comparing against the planned groups for each synthesis (item #5)). | Method: Data synthesis |
|  | 13b | Describe any methods required to prepare the data for presentation or synthesis, such as handling of missing summary statistics, or data conversions. | Method: Data synthesis |
|  | 13c | Describe any methods used to tabulate or visually display results of individual studies and syntheses. | Method: Data synthesis |
|  | 13d | Describe any methods used to synthesize results and provide a rationale for the choice(s). If meta-analysis was performed, describe the model(s), method(s) to identify the presence and extent of statistical heterogeneity, and software package(s) used. | Method: Data synthesis |
|  | 13e | Describe any methods used to explore possible causes of heterogeneity among study results (e.g. subgroup analysis, meta-regression). | Method: Data synthesis |
|  | 13f | Describe any sensitivity analyses conducted to assess robustness of the synthesized results. | Method: Data synthesis |
| Reporting bias assessment | 14 | Describe any methods used to assess risk of bias due to missing results in a synthesis (arising from reporting biases). | Results: Quality appraisal of studies measuring HSUV in older women |
| Certainty assessment | 15 | Describe any methods used to assess certainty (or confidence) in the body of evidence for an outcome. | Not report |
| **RESULTS** | | |  |
| Study selection | 16a | Describe the results of the search and selection process, from the number of records identified in the search to the number of studies included in the review, ideally using a flow diagram. | Results: Selection of studies |
|  | 16b | Cite studies that might appear to meet the inclusion criteria, but which were excluded, and explain why they were excluded. | Results: Selection of studies |
| Study characteristics | 17 | Cite each included study and present its characteristics. | Results: Study characteristics |
| Risk of bias in studies | 18 | Present assessments of risk of bias for each included study. | Results: Quality appraisal of studies measuring HSUV in older women |
| Results of individual studies | 19 | For all outcomes, present, for each study: (a) summary statistics for each group (where appropriate) and (b) an effect estimate and its precision (e.g. confidence/credible interval), ideally using structured tables or plots. | Results: Study characteristics |
| Results of syntheses | 20a | For each synthesis, briefly summarise the characteristics and risk of bias among contributing studies. | Results: Regression analysis |
|  | 20b | Present results of all statistical syntheses conducted. If meta-analysis was done, present for each the summary estimate and its precision (e.g. confidence/credible interval) and measures of statistical heterogeneity. If comparing groups, describe the direction of the effect. | Results: Regression analysis |
|  | 20c | Present results of all investigations of possible causes of heterogeneity among study results. | Not reported |
|  | 20d | Present results of all sensitivity analyses conducted to assess the robustness of the synthesized results. | Not reported |
| Reporting biases | 21 | Present assessments of risk of bias due to missing results (arising from reporting biases) for each synthesis assessed. | Not reported |
| Certainty of evidence | 22 | Present assessments of certainty (or confidence) in the body of evidence for each outcome assessed. | Not reported |
| **DISCUSSION** | | |  |
| Discussion | 23a | Provide a general interpretation of the results in the context of other evidence. | Discussion |
|  | 23b | Discuss any limitations of the evidence included in the review. | Discussion |
|  | 23c | Discuss any limitations of the review processes used. | Discussion |
|  | 23d | Discuss implications of the results for practice, policy, and future research. | Discussion |
| **OTHER INFORMATION** | | |  |
| Registration and protocol | 24a | Provide registration information for the review, including register name and registration number, or state that the review was not registered. | Method |
|  | 24b | Indicate where the review protocol can be accessed, or state that a protocol was not prepared. | Method |
|  | 24c | Describe and explain any amendments to information provided at registration or in the protocol. | Method |
| Support | 25 | Describe sources of financial or non-financial support for the review, and the role of the funders or sponsors in the review. | End of the article |
| Competing interests | 26 | Declare any competing interests of review authors. | End of the article |
| Availability of data, code and other materials | 27 | Report which of the following are publicly available and where they can be found: template data collection forms; data extracted from included studies; data used for all analyses; analytic code; any other materials used in the review. | Appendices |

*From:*  Page MJ, McKenzie JE, Bossuyt PM, Boutron I, Hoffmann TC, Mulrow CD, et al. The PRISMA 2020 statement: an updated guideline for reporting systematic reviews. BMJ 2021;372:n71. doi: 10.1136/bmj.n71 For more information, visit: <http://www.prisma-statement.org/>

**Appendix 2. Search Strategy for Databases**

1. Embase from 2009 January to 2021 22 September

| No | Searches | No |
| --- | --- | --- |
| 1 | quality adjusted life year/ | 28166 |
| 2 | (quality adjusted or adjusted life year$).ti,ab,sh. | 26226 |
| 3 | (qaly$ or qald$ or qale$ or qtime$).ti,ab,sh. | 21317 |
| 4 | (illness state$1 or health state$1).ti,ab,sh. | 11884 |
| 5 | (hui or hui1 or hui2 or hui3).ti,ab,sh. | 2429 |
| 6 | (multiattribute$ or multi attribute$).ti,ab,sh. | 1192 |
| 7 | (utility adj3 (score$1 or valu$ or health$ or cost$ or measur$ or disease$ or mean or gain or gains or index$)).ti,ab,sh. | 25144 |
| 8 | utilities.ti,ab,sh. | 12241 |
| 9 | (eq-5d or eq5d or eq-5 or eq5 or euro qual or euroqual or euro qual5d or euroqual5d or euro qol or euroqol or euro qol5d or euroqol5d or euro quol or euroquol or euro quol5d or euroquol5d or eur qol or eurqol or eur qol5d or eur qol5d or eur?qul or eur?qul5d or euro$ quality of life or European qol).ti,ab,sh. | 23018 |
| 10 | (euro$ adj3 (5 d or 5d or 5 dimension$ or 5dimension$ or 5 domain$ or 5domain$)).ti,ab,sh. | 6750 |
| 11 | (sf36$ or sf 36$ or sf thirtysix or sf thirty six).ti,ab,sh. | 39281 |
| 12 | (time trade off$1 or time tradeoff$1 or tto or timetradeoff$1).ti,ab,sh. | 2894 |
| 13 | 1 or 2 or 3 or 4 or 5 or 6 or 7 or 8 or 9 or 10 or 11 or 12 | 123690 |
| 14 | breast tumor/ | 83575 |
| 15 | exp breast/ or exp breast disease/ | 638553 |
| 16 | exp malignant neoplasm/ | 3567680 |
| 17 | (cancer$ adj3 breast$).tw. | 436322 |
| 18 | (neoplas$ adj3 breast$).tw. | 3454 |
| 19 | (carcinoma$ adj3 breast$).tw. | 53176 |
| 20 | (adenocarcinoma$ adj3 breast$).tw. | 5276 |
| 21 | (tumour$ adj3 breast$).tw. | 8365 |
| 22 | (tumor$ adj3 breast$).tw. | 46012 |
| 23 | (malignan$ adj3 breast$).tw. | 13741 |
| 24 | 15 and 16 | 514234 |
| 25 | 17 or 18 or 19 or 20 or 21 or 22 or 23 | 482462 |
| 26 | 14 or 24 or 25 | 635027 |
| 27 | limit 26 to female | 322144 |
| 28 | premenopause/ | 20761 |
| 29 | 27 not 28 | 316825 |
| 30 | 13 and 29 | 2179 |
| 31 | limit 31 from 2009 to current | 1807 |

1. Ovid MEDLINE(R) and Epub Ahead of Print, In-Process & Other Non-Indexed Citations, Daily and Versions(R) 2009 January to 2021 22 September

| No | Searches | No |
| --- | --- | --- |
| 1 | Quality-Adjusted Life Years/ | 12838 |
| 2 | (quality adjusted or adjusted life year$).ti,ab,kf. | 18164 |
| 3 | (qaly$ or qald$ or qale$ or qtime$).ti,ab,kf. | 11523 |
| 4 | (illness state$1 or health state$1).ti,ab,kf. | 6860 |
| 5 | (hui or hui1 or hui2 or hui3).ti,ab,kf. | 1615 |
| 6 | (multiattribute$ or multi attribute$).ti,ab,kf. | 969 |
| 7 | (utility adj3 (score$1 or valu$ or health$ or cost$ or measur$ or disease$ or mean or gain or gains or index$)).ti,ab,kf. | 16214 |
| 8 | utilities.ti,ab,kf. | 7601 |
| 9 | (eq-5d or eq5d or eq-5 or eq5 or euro qual or euroqual or euro qual5d or euroqual5d or euro qol or euroqol or euro qol5d or euroqol5d or euro quol or euroquol or euro quol5d or euroquol5d or eur qol or eurqol or eur qol5d or eur qol5d or eur?qul or eur?qul5d or euro$ quality of life or European qol).ti,ab,kf. | 12668 |
| 10 | (euro$ adj3 (5 d or 5d or 5 dimension$ or 5dimension$ or 5 domain$ or 5domain$)).ti,ab,kf. | 4481 |
| 11 | (sf36$ or sf 36$ or sf thirtysix or sf thirty six).ti,ab,kf. | 23020 |
| 12 | (time trade off$1 or time tradeoff$1 or tto or timetradeoff$1).ti,ab,kf. | 1979 |
| 13 | quality of life/ and ((quality of life or qol) adj (score$1 or measure$1)).ti,ab,kf. | 12188 |
| 14 | 1 or 2 or 3 or 4 or 5 or 6 or 7 or 8 or 9 or 10 or 11 or 12 | 75568 |
| 15 | exp Breast Neoplasms/ | 299217 |
| 16 | exp Breast/ or exp Breast Diseases/ | 334158 |
| 17 | exp Neoplasms/ | 3409962 |
| 18 | (cancer$ adj3 breast$).tw. | 301974 |
| 19 | (neoplas$ adj3 breast$).tw. | 2887 |
| 20 | (carcinoma$ adj3 breast$).tw. | 41967 |
| 21 | (adenocarcinoma$ adj3 breast$).tw. | 3935 |
| 22 | (tumour$ adj3 breast$).tw. | 5816 |
| 23 | (tumor$ adj3 breast$).tw. | 32148 |
| 24 | (malignan$ adj3 breast$).tw. | 9871 |
| 25 | 16 and 17 | 303516 |
| 26 | 18 or 19 or 20 or 21 or 22 or 23 or 24 | 340170 |
| 27 | 15 or 25 or 26 | 410862 |
| 28 | Premenopause/ | 7909 |
| 29 | limit 27 to female | 293725 |
| 30 | 29 not 28 | 291186 |
| 31 | 14 and 30 | 1380 |
| 32 | limit 31 from 2009 to current | 1215 |

Appendix 3. Quality Appraisal Tool

| **Questions concerning** | |
| --- | --- |
| Data source selection of HSUV used by authors of CUA | |
| S1. | What is (are) the data source(s) of HSUVs? |
| S2. | If HSUVs are derived from the literature, how many references are given? |
| S3. | If derived from the literature, what is (are) the data source(s) of HSUVs? |
| Elicitation of HSUVs used by authors of CUA | |
| E1a. | Is an explanation provided for the choice of technique(s) used to elicit HSUVs? |
| E1b. | Is a comprehensive description provided of technique(s) used to elicit the obtained HSUVs? |
| E2a. | Is an explanation provided for the choice of the population used to elicit HSUVs (i.e., patient, healthcare professional [and type], expert, general population)? |
| E2b. | Is a comprehensive description provided for the population used to elicit HSUVs (i.e., characteristics, size, and nationality)? |
| Use of HSUVs by authors of CUAs: | |
| U1. | Are the HSUVs appropriate with respect to comparability of populations (i.e., diagnosis and disease severity)? |
| U2. | Are the HSUVs appropriate with respect to comparability of countries? |
| U3. | Is the difference between when the CUA was performed and when the HSUVs were elicited less than 10 years? |
| U4. | Do the authors use specific utility values for each health state of the model in the CUA? |
| U5. | Do the authors use only a single source of utility values for each health state of the model in the CUA? |
| U6. | Do the authors use specific utility values for each of the compared interventions in the CUA? |
| U7. | Do the authors use the same HSUVs in the CUA as presented in the original data source? |
| U8. | Do the authors provide a comprehensive description and explanation for the explicit assumptions on the use of the HSUVs in the CUA? |
| U9. | Do the authors report results from a deterministic and probabilistic sensitivity analysis for the HSUVs? |
| U10. | Do the authors discuss the limitations of the data source selection, the elicitation, and the use of HSUVs in the CUA? |

(Note): CUA: cost–utility analysis; E: elicitation; HSUV(s): health-state utility value(s); S: source; U: use

(Source): Nerich et al (2007)

**Appendix 4. Reasons for Excluding Studies during Database Record Screening**

| **Exclusion** | **Reasons** | **Number** |
| --- | --- | --- |
| Condition  (n=301) | Only premenopausal | 10 |
|  | Only metastatic breast cancer | 212 |
|  | Unconfirmed breast cancer | 6 |
|  | Other diseases | 73 |
| Intervention and comparator | Cancer-related symptoms assessment without health state-reported | 173 |
| Outcomes | No original utility reported or from previous literature | 498 |
|  | Unspecified/not clearly specified health states relating to breast cancer | 59 |
|  | Quality of life measured without being synthesised to utility values | 31 |
|  | Only the utility of side effects measured | 2 |
| Study type | Psychometric validation studies | 198 |
|  | Description of health states without interval properties rather than the valuation of health states | 192 |
| Publication | Conference abstract or heading or letters | 652 |
| Language | Language | 3 |

**Appendix 5. Data Extraction of the Mean Utility Values**

| **Study** | **Mean value** | **Standard deviation** | **States** | **Sample size** | **Mean age** | **Instrument** | **Treatment** | **Tariff** | **Measuring time** |
| --- | --- | --- | --- | --- | --- | --- | --- | --- | --- |
| Conner-Spady*, et al.* (2005) [1] | 0.79 | 0.19 | Stable | 45 | 45 | EQ-5D-3L | Surgery +Chemo | UK | < 1year |
| Conner-Spady, et al. (2005) [1] | 0.84 | 0.19 | Stable | 40 | 45 | EQ-5D-3L | Surgery +Chemo | UK | < 1year |
| Conner-Spady, et al. (2005) [1] | 0.84 | 0.13 | Stable | 36 | 45 | EQ-5D-3L | Surgery +Chemo | UK | > 1 year |
| Conner-Spady, et al. (2005) [1] | 0.89 | 0.13 | Stable | 37 | 45 | EQ-5D-3L | Surgery +Chemo | UK | > 1 year |
| Etikasari*, et al.* (2021) [2] | 0.584 | 0.44 | Advanced | 126 | 59.2 | EQ-5D-5L | Surgery | Indonesian | > 1 year |
| Etikasari*, et al.* (2021) [2] | 0.768 | 0.19 | Progression | 126 | 59.2 | EQ-5D-5L | Surgery | Indonesian | > 1 year |
| Etikasari*, et al.* (2021) [2] | 0.871 | 0.1 | Stable | 126 | 59.2 | EQ-5D-5L | Surgery | Indonesian | < 1year |
| Freedman*, et al.* (2010) [3]^*^ | 0.89 | 0.33 | Stable | 1050 | 50 | EQ-5D-3L | Surgery +Radio | USA | > 1 year |
| Freedman*, et al.* (2010) [3]^*^ | 0.9 | 0.66 | Stable | 1050 | 50 | EQ-5D-3L | Surgery +Radio | USA | > 1 year |
| Kimman*, et al.* (2009) [4] | 0.72 | 0.29 | Progression | 23 | 55.8 | EQ-5D-3L | Surgery without specified adjuvant | UK | > 1 year |
| Kimman, et al. (2009) [4] | 0.73 | 0.18 | Progression | 14 | 55.8 | EQ-5D-3L | Surgery without specified adjuvant | UK | > 1 year |
| Kimman, et al. (2009) [4] | 0.71 | 0.2 | Stable | 72 | 55.8 | EQ-5D-3L | Surgery without specified adjuvant | UK | > 1 year |
| Kimman, et al. (2009) [4] | 0.78 | 0.14 | Stable | 28 | 55.8 | EQ-5D-3L | Surgery without specified adjuvant | UK | > 1 year |
| Kimman, et al. (2009) [4] | 0.82 | 0.21 | Stable | 55 | 55.8 | EQ-5D-3L | Surgery without specified adjuvant | UK | > 1 year |
| Lidgren*, et al.* (2007) [5] | 0.685 | 0.34 | Advanced | 345 | 57 | EQ-5D-3L | Unspecified | UK | > 1 year |
| Lidgren, et al. (2007) [5] | 0.779 | 0.12 | Progression | 345 | 57 | EQ-5D-3L | Unspecified | UK | > 1 year |
| Lidgren, et al. (2007) [5] | 0.779 | 0.2 | Progression | 345 | 57 | EQ-5D-3L | Unspecified | UK | > 1 year |
| Lidgren, et al. (2007) [5] | 0.696 | 0.535 | Stable | 345 | 57 | EQ-5D-3L | Unspecified | UK | < 1year |
| Sattar*, et al.* (2019) [6] | 0.78 | 0.15 | Stable | 18 | 75.3 | EQ-5D-3L | Surgery +Chemo | Canada | < 1year |
| Sattar*, et al.* (2019) [6] | 0.82 | 0.29 | Stable | 24 | 74.7 | EQ-5D-3L | Surgery +Chemo | Canada | < 1year |
| Sattar*, et al.* (2019) [6] | 0.82 | 0.27 | Stable | 21 | 74.7 | EQ-5D-3L | Surgery +Chemo | Canada | < 1year |
| Sattar*, et al.* (2019) [6] | 0.83 | 0.22 | Stable | 12 | 75.3 | EQ-5D-3L | Surgery +Chemo | Canada | < 1year |
| Tanaka*, et al.* (2019) [7] | 0.757 | 0.2 | Stable | 19 | 53.4 | EQ-5D-3L | Surgery +Chemo | UK | < 1year |
| Tanaka*, et al.* (2019) [7] | 0.791 | 0.16 | Stable | 19 | 53.4 | EQ-5D-3L | Surgery +Chemo | UK | < 1year |
| Tanaka*, et al.* (2019) [7] | 0.831 | 0.13 | Stable | 19 | 53.4 | EQ-5D-3L | Surgery +Chemo | UK | < 1year |
| Tanaka*, et al.* (2019) [7] | 0.882 | 0.14 | Stable | 19 | 56.7 | EQ-5D-3L | Surgery +Chemo | UK | < 1year |
| Tanaka*, et al.* (2019) [7] | 0.883 | 0.13 | Stable | 19 | 56.7 | EQ-5D-3L | Surgery +Chemo | UK | < 1year |
| Tanaka*, et al.* (2019) [7] | 0.921 | 0.12 | Stable | 19 | 56.7 | EQ-5D-3L | Surgery +Chemo | UK | < 1year |
| Wang*, et al.* (2018) [8] | 0.686 | 0.57 | Advanced | 224 | 50.7 | EQ-5D-3L | Unspecified | China | > 1 year |
| Wang*, et al.* (2018) [8] | 0.774 | 0.4 | Advanced | 556 | 49.1 | EQ-5D-3L | Unspecified | China | > 1 year |
| Wang*, et al.* (2018) [8] | 0.789 | 0.18 | Stable | 1234 | 49.8 | EQ-5D-3L | Unspecified | China | > 1 year |
| Wang*, et al.* (2018) [8] | 0.792 | 0.34 | Stable | 498 | 49.1 | EQ-5D-3L | Unspecified | China | > 1 year |
| Williams (2011) [9] | 0.78 | 0.2 | Stable | 128 | 72.3 | EQ-5D-3L | Surgery + Endocrine + Radio | UK | < 1year |
| Williams (2011) [9] | 0.77 | 0.2 | Stable | 126 | 72.8 | EQ-5D-3L | Surgery + Endocrine | UK | < 1year |
| Yang*, et al.* (2020) [10] | 0.85 | 0.45 | Advanced | 43 | 51.2 | EQ-5D-5L | Unspecified | China | > 1 year |
| Yang*, et al.* (2020) [10] | 0.94 | 0.17 | Progression | 258 | 52.7 | EQ-5D-5L | Unspecified | China | < 1year |
| Yang*, et al.* (2020) [10] | 0.92 | 0.63 | Progression | 20 | 49.9 | EQ-5D-5L | Unspecified | China | > 1 year |
| Yang*, et al.* (2020) [10] | 0.89 | 0.24 | Stable | 125 | 51.4 | EQ-5D-5L | Unspecified | China | > 1 year |
| Yousefi*, et al.* (2016) [11] | 0.552 | 0.23 | Advanced | 24 | 46.7 | EQ-5D-3L | Unspecified | UK | > 1 year |
| Yousefi*, et al.* (2016) [11] | 0.73 | 0.22 | Progression | 71 | 46.7 | EQ-5D-3L | Unspecified | UK | > 1 year |
| Yousefi*, et al.* (2016) [11] | 0.718 | 0.14 | Progression | 15 | 46.7 | EQ-5D-3L | Unspecified | UK | > 1 year |
| Yousefi*, et al.* (2016) [11] | 0.674 | 0.2 | Stable | 48 | 46.7 | EQ-5D-3L | Unspecified | UK | < 1year |
| Yu*, et al.* (2018) [12] | 0.862 | 0.11 | Stable | 39 | 48.9 | EQ-5D-3L | Surgery +Chemo | Korea | < 1year |
| Yu*, et al.* (2018) [12] | 0.902 | 0.08 | Stable | 92 | 49.1 | EQ-5D-3L | Surgery +Chemo | Korea | < 1year |
| Yu*, et al.* (2018) [12] | 0.909 | 0.09 | Stable | 149 | 49.5 | EQ-5D-3L | Surgery +Chemo | Korea | > 1 year |
| Yu*, et al.* (2018) [12] | 0.919 | 0.09 | Stable | 226 | 53.6 | EQ-5D-3L | Surgery +Chemo | Korea | > 1 year |
| Yu*, et al.* (2018) [12] | 0.924 | 0.08 | Stable | 180 | 49.5 | EQ-5D-3L | Surgery +Chemo | Korea | > 1 year |
| Zigman*, et al.* (2020) [13] | 0.62 | 0.34 | Advanced | 32 | 54 | EQ-5D-3L | Unspecified | UK | > 1 year |
| Zigman*, et al.* (2020) [13] | 0.78 | 0.16 | Progression | 49 | 56.2 | EQ-5D-3L | Unspecified | UK | > 1 year |
| Zigman*, et al.* (2020) [13] | 0.85 | 0.17 | Stable | 33 | 44.7 | EQ-5D-3L | Unspecified | UK | > 1 year |

**References**

1. Conner-Spady, B.L.*, et al.*, *A longitudinal prospective study of health-related quality of life in breast cancer patients following high-dose chemotherapy with autologous blood stem cell transplantation.* Bone Marrow Transplant, 2005. **36**(3): p. 251-9.

2. Etikasari, R.*, et al.*, *Health related quality of life among postmenopausal woman with hormone responsive HER2- breast cancer in Indonesia.* J Basic Clin Physiol Pharmacol, 2021. **32**(4): p. 561-565.

3. Freedman, G.M.*, et al.*, *Health states of women after conservative surgery and radiation for breast cancer.* Breast Cancer Res Treat, 2010. **121**(2): p. 519-26.

4. Kimman, M.L.*, et al.*, *Responsiveness of the EQ-5D in breast cancer patients in their first year after treatment.* Health Qual Life Outcomes, 2009. **7**: p. 11.

5. Lidgren, M.*, et al.*, *Health related quality of life in different states of breast cancer.* Qual Life Res, 2007. **16**(6): p. 1073-81.

6. Sattar, S.*, et al.*, *Health status, emergency department visits, and oncologists' feedback: An analysis of secondary endpoints from a randomized phase II geriatric assessment trial.* J Geriatr Oncol, 2019. **10**(1): p. 169-174.

7. Tanaka, K.*, et al.*, *Cost utility analysis of pharmacist counseling care for breast cancer chemotherapy outpatients.* Pharmazie, 2019. **74**(7): p. 439-442.

8. Wang, L.*, et al.*, *Health-related quality of life and utility scores of patients with breast neoplasms in China: A multicenter cross-sectional survey.* Breast, 2018. **39**: p. 53-62.

9. Williams, L. *et al.*, *A randomised controlled trial of post-operative radiotherapy following breast-conserving surgery in a minimum-risk population. Quality of life at 5 years in the PRIME trial.* Health Technol Assess, 2011. **15**(12): p. 1-57.

10. Yang, Q., X. Yu, and W. Zhang, *Health variations among breast-cancer patients from different disease states: evidence from China.* BMC Health Serv Res, 2020. **20**(1): p. 1033.

11. Yousefi, M.*, et al.*, *Comparison of SF-6D and EQ-5D Scores in Patients With Breast Cancer.* Iran Red Crescent Med J, 2016. **18**(5): p. e23556.

12. Yu, J.*, et al.*, *Uneven recovery patterns of compromised health-related quality of life (EQ-5D-3 L) domains for breast Cancer survivors: a comparative study.* Health Qual Life Outcomes, 2018. **16**(1): p. 143.

13. Zigman, T.*, et al.*, *Defining health-related quality of life in localized and advanced stages of breast cancer - the first step towards hereditary cancer genetic counseling.* Acta Clin Croat, 2020. **59**(2): p. 209-215.
